# High rates of long‐term progression in HIV‐1‐positive elite controllers

**DOI:** 10.1002/jia2.25675

**Published:** 2021-02-22

**Authors:** Maria Borrell, Irene Fernández, Flor Etcheverrry, Ainoa Ugarte, Montserrat Plana, Lorna Leal, Felipe García

**Affiliations:** ^1^ Infectious Diseases Department Hospital Clínic IDIBAPS University of Barcelona Spain; ^2^ Retrovirology and Viral Immunopathology AIDS Research Group IDIBAPS, Hospital Clinic University of Barcelona Barcelona Spain

**Keywords:** Elite controllers, HIV‐1, progression, outcome

## Abstract

**Introduction:**

Elite controllers (EC) are a rare group of HIV‐1‐positive individuals who suppress viral loads (VL) to undetectable levels with elevated CD4 T‐cell counts in the absence of ART. While rates of short‐ and mid‐term progression have been described in these patients, few studies have focused on their long‐term outcome This study aims to describe the virological and immunological behaviour in a cohort of elite controllers followed up for a median of 17 years in the University Hospital, and to identify factors that may be related to disease progression.

**Methods:**

We conducted a descriptive, prospective and single‐centre study of all HIV‐positive adults recorded in the University Hospital database who met the definition criteria for EC. EC were defined as patients having two consecutive undetectable VL without ART for at least one year. Patients were followed from baseline up to December 2019, to the development of a progression event (loss of VL control, CD4+ T cell decline, AIDS or death) or to the censoring date (lost to follow‐up or initiation of ART). Predictive models of progression were calculated.

**Results:**

Fifty‐nine EC were identified with a median follow‐up of 17 years contributing 1033 PYFU. The median (95% CI) time duration from HIV‐1 diagnosis to disease progression was four (1.7 to 6.3) years. Forty‐nine (83%) presented progression to the composite end‐point, 44 (74.6%) lost viral control, 39 (66.1%) lost immunological control, two developed AIDS and two died. Only 10 patients (16.9%) did not show progression of any kind. Independent predictors of virological progression were sexual risk of HIV‐1 acquisition and VL blips during the first year of follow‐up (baseline). The only independent predictor detected for progression to a composite end‐point was VL blips during the first year of follow‐up (baseline).

**Conclusions:**

The rate of long‐term progression in EC was very high. Only a minority of patients did not show clinical progression after a median of 17 years of follow‐up. These results should be taken in account when considering EC as a model of HIV‐1 remission.

## 1 INTRODUCTION

The success of combination antiretroviral therapy (ART) in relation to survival and quality of life in patients with HIV has led HIV researchers to pursue new avenues toward finding a cure. Although the ultimate elimination of HIV reservoirs remains a distant goal, the existence of elite controllers (EC) has guided the creation of a plausible model of functional cure. Elite controllers are HIV‐1‐positive individuals who spontaneously suppress viraemia to undetectable viral loads (< 50 copies/mL) while maintaining elevated CD4 cell counts without receiving antiretroviral therapy. These patients are rare, representing only 0.2 ‐ 0.5% of HIV‐1‐positive cases [1].

EC follow a heterogeneous pattern of evolution and their clinical outcomes are diverse. Among them, one subgroup displays prolonged control of clinical progression without ART. Previous reports have described several selected cases of durable control over HIV‐1 replication without ART, who are probably the closest to achieving spontaneous functional HIV‐1 cure [2, 3, 4]. In a significant number of individuals, however, progression is less positive. In a cohort study comprising 204 elite controllers, 23% showed a loss of viral control over time [5]. Factors related to virological failure have not yet been well established, due to the diversity of study designs and the lack of longitudinal studies [6]. However, some cohorts have shown evidence of certain predictors [5], and proteomic studies have stressed the potential of biomarkers, which have been associated with higher levels of inflammation, transendothelial migration and coagulation [7, 8, 9, 10]. Understanding the process of viral control would enable practitioners to offer appropriate medical care to those EC who will undergo virological failure in the near future, and may also guide the design of strategies for developing a functional cure.

Some elite controllers experience non‐AIDS defining events, which are the main cause of morbidity and mortality among HIV‐positive patients on ART [11]. The prevalence of atherosclerosis is increased in EC compared to HIV‐negative controls, as are markers of immune activation [12]. However, some authors report similar rates of non‐AIDS events in EC and immunopreserved HIV‐positive patients [13], whereas others have found that EC showed higher rates of hospitalization than patients on ART [14]. In general, more standardized research projects are needed in order to understand the factors associated with the loss of EC status, as well as the role of spontaneous control of viraemia in the development of non‐AIDS events.

Although several studies have analysed the rate of progression in EC, most of them have reported data with short‐ or mid‐term follow‐up only [5, 15, 16, 17, 18, 19]. This study aims to describe the virological and immunological behaviour of a cohort of elite controllers followed up for a median of 17 years at the University Hospital and to identify factors that may be related to disease progression.

## 2 METHODS

### 2.1 Patients and data collection

We conducted a descriptive, prospective, single‐centre study of all HIV‐positive adults recorded in the University Hospital database who met the definition criteria for elite controllers. Elite controller status was described as the maintenance of at least two consecutive undetectable plasma viral loads (under 50 copies/mL) during a period no shorter than 12 months, in the absence of any ART. All these patients were identified in the chronic phase of infection. The criteria used are those included in the most frequent definition of EC in the literature [20]. In addition, a CD4 T cell count above 500 cells/mm^3^ was also considered as a definition criterion. Assessing old HIV‐1 viral load determinations with a detection level below 200, 49 and 36 copies/mL, patients with viral load (VL) under this cutoff point were also considered EC. All patients were tested three or four times during the first year of follow‐up. Thereafter, viral load and CD4 T cell count determinations were performed every six months. Written consent and all necessary data were prospectively and routinely collected in this cohort using predefined questionnaires so as to allow the identification of HIV‐1 controllers. Each participant signed an informed consent document. The programme was approved by the Institutional Review Boards of the Hospital Clínic of Barcelona.

EC were followed from their initial visit to the Hospital Clínic since 1987 to December 2019, to loss to follow‐up or initiation of ART, or to the development of a progression event. Progression events were as follows. Virological: defined by Lambotte O et al. [21] as a failure to maintain more than 90% of HIV‐1‐RNA determinations below the VL thresholds used to define controllers, throughout follow‐up and without ART; immunological: a reduction in CD4+ cell count below 500 cells/mm^3^; and clinical: defined as a new AIDS event (grade C according to the Centers for Disease Control and Prevention) or death. The combined endpoint of HIV disease progression was defined as the presence of at least one of these virological, immunological or clinical progression outcomes.

### 2.2 Statistical methodology

For the demographic description, characteristics of the study population were reported as median [interquartile range: IQR]. Models for predicting baseline markers associated with virological, immunological or combined HIV‐1 disease progression included all the available baseline characteristics of the patients: age, sex, risk for HIV‐1 acquisition, hepatitis C virus (HCV) serology, HCV cure during the follow‐up (defined as a sustained virological response) or no detectable HCV replication after 12 weeks of treatment, nadir CD4+ T‐cell count, zenith viral load (VL), baseline CD4+ cell count and baseline VL defined as the first measurement during follow‐up at our centre, and total years of follow‐up. The years of follow‐up were estimated using the date of diagnosis as the starting point.

A univariate analysis by Kaplan–Meier estimates, log‐rank tests and proportional hazard models was performed to analyse the influence of the different variables on the time to study end‐points (time to the increase in VL above detectable levels, time to the decline of CD4+ cell counts below 500 cells/mm^3^ or time to combined progression, involving an AIDS‐defining event, death, starting ART, and virological or CD4 progression). To calculate time to combined progression, the limiting factor was set as the first of the progression events to occur. Subsequently, potential determinants of clinical events were studied using Cox regression models. The significant variables selected in the univariate analysis were included in the multivariate analysis using a stepwise forward (Wald Chi‐squared test) method. All statistical analyses were performed using SPSS software version 25 (SPSS Inc., Chicago, IL, USA).

## 3 RESULTS

### 3.1 Characteristics of participants

Among the 863 patients included in the first search for two undetectable viral loads without ART in the Hospital Clínic HIV database, 59 elite controllers were identified. Patients were followed up for a median of 17 years (IQR 9 to 25), thus contributing 1033 PYFU. The main characteristics of the EC in this study are summarized in Table 1. The median (95% CI) time from HIV‐1 diagnosis to disease progression was 4 (1.7 to 6.3) years. As regards disease development, 49 patients (83%) experienced progression, of whom 39 (66.1%) presented a CD4 cell count drop under 500 cells/mm^3^, 44 (74.6%) lost virological control and two (3.4%) died. As various VL tests and detection limits had been used, we standardized the blipping using 200 copies/mL as the definition in a sensitivity analysis, which confirmed the results (data not shown). In the two patients who died, the cause of death was cirrhosis in one and unknown in the other. Of the 44 patients who experienced virological failure, 36 (81.9%) also presented concomitant immunological failure, two of whom presented AIDS‐defining events (one pulmonary tuberculosis and the other lymph node tuberculosis). Only 10 patients (16.9%) did not show progression of any kind during the follow‐up. Fifty percent of the patients without progression were males with a median (IQR) age of 55 years old (40 to 60), 60% were coinfected by HCV, and the medians (IQR) of baseline and nadir CD4 T cell count were 872 (678 to 1066) and 736 (524 to 835) respectively. In these patients the median (IQR) length of follow‐up was 20 years (5 to 29); five had been followed up for more than 25 years (range 25 to 35 years).

**Table 1:** Demographic characteristics of the EC cohort in this study

Variable	Elite controllers
N	59
Age^a^	38 (31 to 44)
Sex	
Male	35 (59.3%)
Female	24 (40.7%)
Risk for HIV acquisition	
Parenteral	31 (52.5%)
MSM	15 (25.4%)
HET	11 (18.6%)
Unknown	2 (3.4%)
HCV	
Negative serology	26 (44%)
Positive serology	33 (55.9%)
Cured	18 (30.5%)
Not cured	15 (25.4%)
Baseline CD4^+^ T cells/mm^a^	699 (544 to 864)
Nadir CD4^+^ T cells/mm^a^	414 (275 to 536)
Zenith viral load (log_10_ copies/mL)^b^	3.05 (0.13)
Years of follow‐up^a^	17 (9 to 25)

HCV, Hepatitis C Virus evaluated at baseline; HET: Heterosexual; MSM, Men who have sex with men.

^a^ Median (Interquartile Range)

^b^ mean (Standard Error).

### 3.2 Virological progression

**Figure 1:**
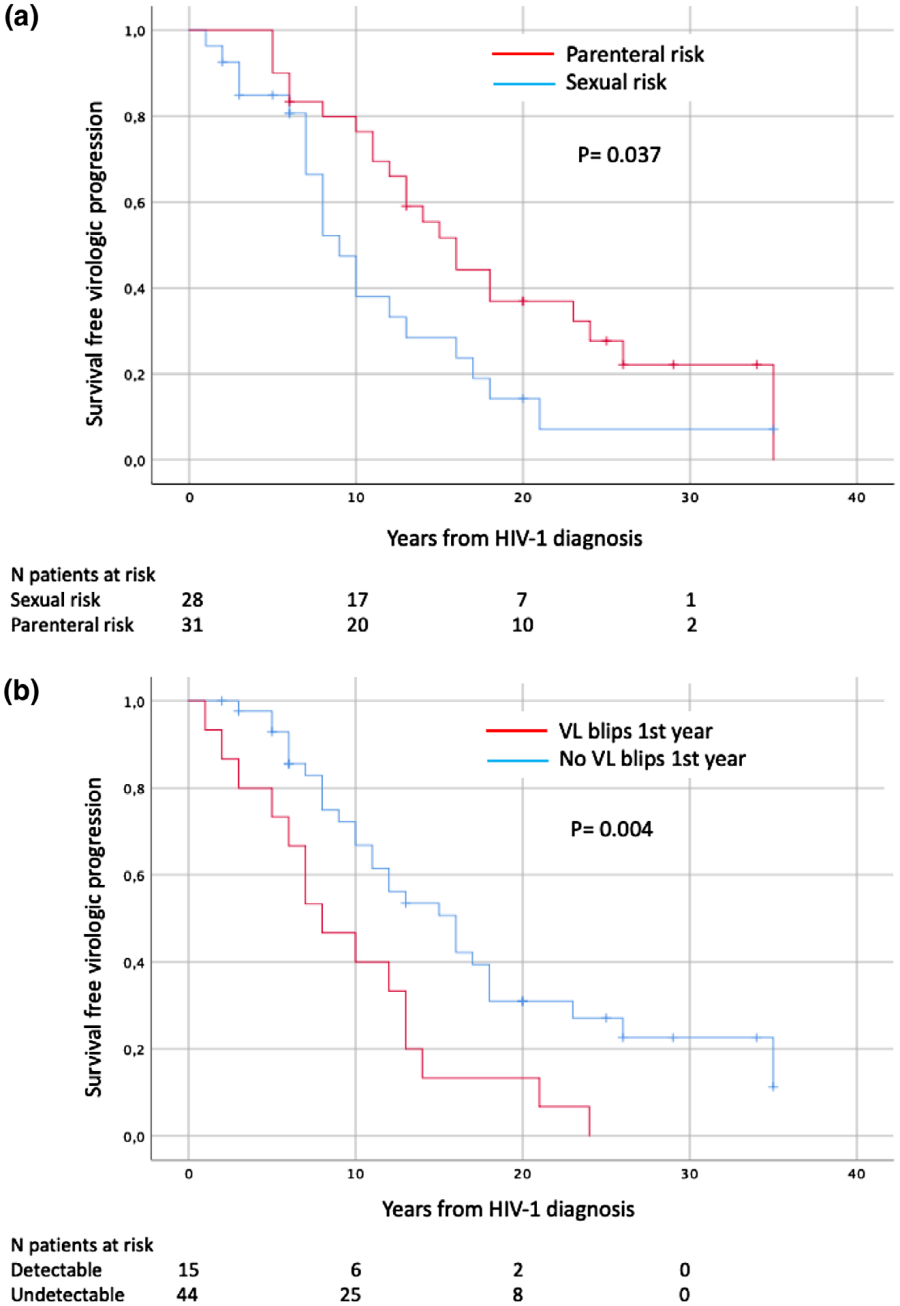
Kaplan–Meier plot of survival free of virological progression according to **(a)** Risk of HIV‐1 acquisition, **(b)** Viral load blips 1st year

The univariate analysis reflected that the age below the median (≤38 years old), parenteral group risk for HIV‐1 acquisition, HCV infection, baseline CD4+ T cell levels above the median and the absence of viral load blips in the first year of follow‐up (baseline) had a significant impact on virological progression. All these variables were associated with a longer maintenance of undetectable viral load levels. Nadir CD4+ T cell levels above the median showed a trend towards persistent viral control. In the multivariate analysis, variables which proved to be independent predictors of VL progression were sexual group risk for HIV‐1 acquisition [HR 2.2 (1.1 to 4.2)] and the presence of viral load blips in the first year of follow‐up (baseline) [HR of 2.1 (1.1 to 4.0)]. All the results are displayed in Tables 2 and 3, and Kaplan–Meier diagrams for independent predictors are shown in Figure 1.

**Table 2:** Univariate analysis for variables that predict persistent control regarding virological *p* and immunological progression

Variable	Years to viral failure^a^	*p* value	Years to CD4 failure^a^	*p* value
Age				
≤38	18 (13 to 23)	0.007	9.5 (1.9 to 17)	0.13
>38	11 (7 to 15)		5.5 (1.8 to 9.2)	
Sex				
Male	12 (7.7 to 16.3)	0.56	7.5 (0 to 15.7)	0.76
Female	13 (8.6 to 17.4)		4 (0.7 to 7.3)	
Risk for HIV acquisition				
Parenteral	16 (12.7 to 19.3)	0.037	5.5 (2.8 to 8.2)	0.96
MSM	8 (6.9 to 9.1)		4 (0 to 8.6)	
HET	12 (7.6 to 16.4)		9.5 (0.8 to 18.2)	
HCV				
Negative serology	8 (5.2 to 10.8)	0.007	7.5 (1.5 to 13.5)	0.77
Positive serology	15 (11 to 18.9)		5.5 (2.1 to 8.9)	
HCV cure				
Cured	16 (7.7 to 24.3)	0.8	4 (0.9 to 7)	0.06
Not cured	14 (10.8 to 17.2)		6 (2.1 to 14)	
Nadir CD4+ T cells				
<414	12 (9.3 to 14.7)	0.12	N/A	N/A
>414	14 (8.2 to 19.8)			
Baseline CD4+ T cells				
<699	10 (6.7 to 13.4)	0.035	N/A	N/A
>700	16 (11.7 to 20.3)			
Viral load blips 1st year				
No	16 (11.4 to 20.6)	0.004	7.5 (0.9 to 14.1)	0.1
Yes	8 (4.2 to 11.8)		3 (0 to 6.2)	

A univariate analysis using Kaplan–Meier estimates and log‐rank tests was performed. HCV, Hepatitis C Virus evaluated at baseline; HET, Heterosexual; MSM, Men who have sex with men.

^a^ Estimated median (95% confidence interval).

**Table 3:** Univariate and multivariate analysis for predictors of combined HIV persistent control and independent predictors of persistent virological and combined control respectively

Univariate analysis				
Variable	Years to combined progression^a^	PYFU	Events (N)/total	*p* value
Age				
≤38	4 (0.7 to 7.3)	633	23/31	0.09
>38	4 (1.6 to 6.4)	400	25/28	
Sex				
Male	4 (0.7 to 7.3)	576	30/35	0.78
Female	4 (0.2 to 7.8)	457	18/24	
Risk for HIV acquisition				
Parenteral	5.5 (3.5 to 7.5)	611	24/30	0.15
MSM	3 (0 to 6.1)	213	15/16	
HET	3 (1.6 to 4.4)	182	7/11	
HCV				
Negative serology	3.5 (1.6 to 5.4)	332	22/26	0.1
Positive serology	5 (2.2 to 7.8)	701	26/33	
HCV cure				
Cured	4 (0.9 to 7)	450	16/18	0.16
Not cured	11 (0 to 24.8)	251	10/15	
Viral load blips 1st year				
No	6 (1.4 to 10.6)	824	33/44	0.003
Yes	2 (1 to 2.9)	209	15/15	

Multivariate analysis				
Independent predictors	Hazard Ratio (95% CI)			*p* value
Virological progression				
Sexual risk for HIV acquisition	2.2 (1.1 to 4.2)			0.020
Viral load blips 1st year	2.1 (1.1 to 4.0)			0.026
Combined progression				
Viral load blips 1st year	2.5 (1.3 to 4.6)			0.005

A univariate analysis using Kaplan–Meier estimates and log‐rank tests was performed. Potential determinants of clinical events in the multivariate analysis were analysed using Cox regression models. HCV, Hepatitis C Virus evaluated at baseline; HET, Heterosexual; MSM, Men who have sex with men; PYFU, Person‐years of follow‐up.

^a^ Estimated median (Interquartile Range).

### 3.3 Immunological progression

Regarding the loss of immunological control over time, patients with cured HCV infection showed a propensity for an earlier CD4+ reduction. In addition, there was a trend towards a slower decline in immunological control in patients with the absence of viral load blips in the first year of follow‐up (baseline). No independent risk factors were detected for immunological progression. Six individuals with only one sporadic drop in CD4+ T cell levels under 500 cells/mm^3^ met our criteria for progression. This isolated CD4+ reduction lasted for less than one year, was not lower than 400 cells/mm^3^ and was not recorded in any of the subsequent determinations. Removal of these six patients from the progression group reduced the immunological failure rate to 55.9%, closer to the 48% reported in the literature [22]. The results are summarized in Table 2.

### 3.4 Combined progression

**Figure 2:**
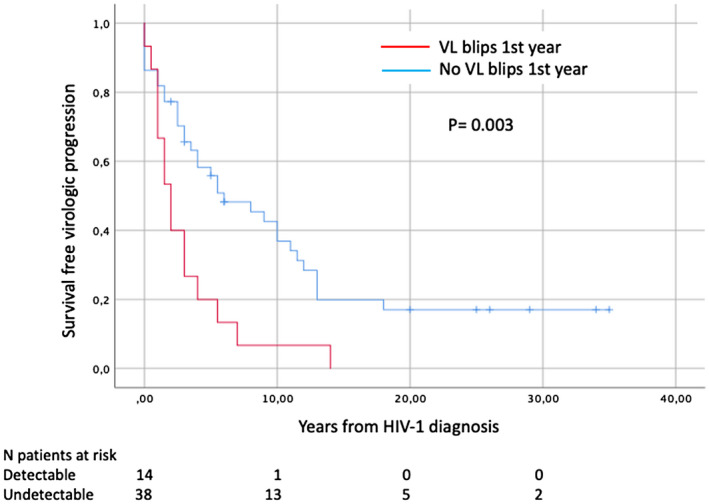
Kaplan–Meier plot of survival free of combined progression concerning baseline viral load

When combining virological and immunological failure rates, a significant gain in progression‐free years was found in patients with no viral load blips in the first year of follow‐up (baseline). Ultimately, the only variable that proved to be an independent predictor for progression after Cox regression was the presence of viral load blips in the first year of follow‐up (baseline) [OR 2.5 (95% CI 1.3 to 4.6)] showing that patients with viral load blips in the first year of follow‐up (baseline) were more likely to present progression of HIV infection. Specific data are shown in Table 3 and the Kaplan–Meier graph regarding baseline viral load as a predictor is shown in Figure 2.

## 4 DISCUSSION

In this study, we outline the main demographic characteristics of elite controllers at the University Hospital, the evolution of their viral, immunological and combined infection control, and also the evolution of the variables that predicted disease progression. In our cohort, rates of loss of spontaneous HIV‐1 control due to an increase in viral load or a reduction in CD4+ T cell count were notably higher than in previous studies, even though we used the same definition for elite control. While one study report incidence rates for viral and combined progression of 24% and 53%, respectively, with a median of 7 years of follow‐up [5], the rates in our patients were 74.6% and 83% after a median of 17 years of follow‐up. In previous studies that used alternative definitions, incidence of virological failure for EC was around 15% [17] after a median of 13 years of follow‐up. Regarding immunological progression, the rate in our patients was 66.1%, which is comparable to the 48% reported in the literature [5]. A possible explanation for these differences might be the longer period of follow‐up in our study; others might be the heterogeneity in EC definitions or the use of different thresholds to define progression. Nevertheless, the development rate of AIDS‐defining events remained low (3.4%), in agreement with previous studies [5].

Interestingly, 10 patients (16.9%) did not show progression of any kind and five of these were followed up longer than 25 years. Casado et al. [2] recently defined exceptional EC (EEC) as HIV‐1 subjects who maintain EC characteristics without disease progression for more than 25 years; they found that these individuals simultaneously exhibited ≥ 3 described host protective alleles, low levels of total HIV‐1 DNA without evidence of replication‐competent viruses consistent with high levels of defective genomes, strong cellular HIV‐1‐specific immune responses, and a high poly‐functionality index. Inflammation levels of EEC were similar to HIV‐1‐negative donors. They also showed a notable lack of viral evolution and an eightfold lower genetic diversity in the env gene than other EC. These EEC may represent cases of spontaneous functional HIV‐1 cure and deserve further investigation.

Regarding the variables that predicted persistent viral load control, elite controllers with a sexual risk of HIV‐1 acquisition or with the presence of viral load blips in the first year of follow‐up (baseline) were twice as likely not to maintain long‐term virological control. Previous studies of these variables have reported similar results [5, 18]. It might be speculated that patients with sexual risk of HIV‐1 acquisition have as a higher risk of HIV‐1 superinfection or of acquiring sexually transmitted disease; as suggested by Noel et al [18], this might influence the viral load increase. However, the reason for these differences in virological progression compared with patients with parenteral risk needs further research. If confirmed, the observation that a lack of blipping in the first year was associated with long‐term suppression could be helpful to determine when to initiate an EC on ART.

Our immunological failure rates did not identify any independent risk factors for control maintenance. However, there was a trend for patients with an absence of viral load blips in the first year of follow‐up (baseline) and for those who were not cured of HCV to maintain CD4+ T cell counts above 500 cells/mm^3^ for longer. The latter result can be attributed to the effect of interferon alpha, the first treatment to appear for HCV, which caused a temporary reduction in CD4+ T cell levels and thus affected our results [23].

With regard to the combined loss of EC status the only independent risk factor observed was the absence of viral load blips in the first year of follow‐up (baseline). Patients with undetectable viral loads since the beginning of follow‐up had an OR of 2.5 (1.3 to 4.6) for a longer survival time until all‐cause progression. A longer period of viral suppression was related to lower rates of disease progression, in agreement with previous studies [1, 5].

Several limitations of this study should be noted. First, because the date of HIV infection was not known, we assumed that patients had been elite controllers since the year of diagnosis. This same assumption was made for patients with the absence of viral load blips in the first year of follow‐up (baseline). Second, despite our rigorous efforts to record CD4+ T cells and VL determinations every six months, not all HIV patients regularly attended their medical appointments. However, as this was not the case for most measurements, we were able to assume that the data collected were representative of six‐month sequences. Finally, with regard to HCV IgG‐positive patients with negative RNA, no distinction between spontaneous clearance or medical treatment was made at the time of analysis. This was the case in four out of 16 cured patients who did not undergo HCV treatment.

## 5 CONCLUSIONS

The frequency of disease progression in elite controllers was much higher than expected. After a median of 17 years of follow‐up, only 17% of patients did not present combined disease progression. Our study raises the question of whether this proportion will decrease further with longer follow‐up further. However, it seems that a small proportion of patients maintain EC status for more than 25 years and may represent cases of spontaneous functional HIV‐1 cure. These data should be taken in account when considering EC as a model of HIV‐1 remission.

## COMPETING INTEREST

The authors do not have a commercial or other association that might pose a conflict of interest.

## AUTHORS’ CONTRIBUTIONS

“MB, IF, FE and AU performed the research. MB, MP, LL and FG designed the research study. MB, FG and FG analysed the data. MB, LL and FG wrote the paper.”

## References

[1] Okulicz JF , Lambotte O . Epidemiology and clinical characteristics of elite controllers. Curr Opin HIV AIDS. 2011;6(3):163–8. 21502920 10.1097/COH.0b013e328344f35e

[2] Casado C , Galvez C , Pernas M , Tarancon‐Diez L , Rodriguez C , Sanchez‐Merino V , et al. Permanent control of HIV‐1 pathogenesis in exceptional elite controllers: a model of spontaneous cure. Sci Rep. 2020;10(1):1902. 32024974 10.1038/s41598-020-58696-y PMC7002478

[3] Canouï E , Lécuroux C , Avettand‐Fenoël V , Gousset M , Rouzioux C , Saez‐Cirion A , et al. A Subset of extreme human immunodeficiency virus (HIV) controllers is characterized by a small HIV blood reservoir and a weak T‐cell activation level. open forum. Infect Dis. 2017;4:ofx064. 10.1093/ofid/ofx064 PMC5450900 28584850

[4] Mendoza D , Johnson SA , Peterson BA , Natarajan V , Salgado M , Dewar RL , et al. Comprehensive analysis of unique cases with extraordinary control over HIV replication. Blood. 2012;119(20):4645–55. 22490332 10.1182/blood-2011-10-381996 PMC3367872

[5] Leon A , Perez I , Ruiz‐Mateos E , Miguel Benito J , Leal M , Lopez‐Galindez C , et al. Rate and predictors of progression in elite and viremic HIV‐1 controllers. AIDS. 2016;30(8):1209–20. 26854807 10.1097/QAD.0000000000001050

[6] Pernas M , Tarancon‐Diez L , Rodriguez‐Gallego E , Gomez J , Prado JG , Casado C , et al. Factors leading to the loss of natural elite control of HIV‐1 infection. J Virol. 2018;92:e01805. 29212942 10.1128/JVI.01805-17 PMC5809746

[7] Rodriguez‐Gallego E , Tarancon‐Diez L , Garcia F , Del Romero J , Benito JM , Alba V , et al. Proteomic profile associated with loss of spontaneous human immunodeficiency virus type 1 elite control. J Infect Dis. 2019;219(6):867–76. 30312441 10.1093/infdis/jiy599

[8] Hunt PW , Brenchley J , Sinclair E , McCune JM , Roland M , Page‐Shafer K , et al. Relationship between T cell activation and CD4+ T cell count in HIV‐seropositive individuals with undetectable plasma HIV RNA levels in the absence of therapy. J Infect Dis. 2008;197(1):126–33. 18171295 10.1086/524143 PMC3466592

[9] Li JZ , Segal FP , Bosch RJ , Lalama CM , Roberts‐Toler C , Delagreverie H , et al. Antiretroviral therapy reduces T‐cell activation and immune exhaustion markers in human immunodeficiency virus controllers. Clin Infect Dis. 2020;70(8):1636–42. 31131858 10.1093/cid/ciz442 PMC7146008

[10] Li JZ , Arnold KB , Lo J , Dugast AS , Plants J , Ribaudo HJ , et al. Differential levels of soluble inflammatory markers by human immunodeficiency virus controller status and demographics. Open Forum. Infect Dis. 2015;2:ofu117. 10.1093/ofid/ofu117 PMC4396431 25884005

[11] Dominguez‐Molina B , Leon A , Rodriguez C , Benito JM , Lopez‐Galindez C , Garcia F , et al. Analysis of non‐AIDS‐defining events in HIV controllers. Clin Infect Dis. 2016;62(10):1304–9. 26936669 10.1093/cid/ciw120

[12] Pereyra F , Lo J , Triant VA , Wei J , Buzon MJ , Fitch KV , et al. Increased coronary atherosclerosis and immune activation in HIV‐1 elite controllers. AIDS. 2012;26(18):2409–12. 23032411 10.1097/QAD.0b013e32835a9950 PMC3660105

[13] Lucero C , Torres B , Leon A , Calvo M , Leal L , Perez I , et al. Rate and predictors of non‐AIDS events in a cohort of HIV‐infected patients with a CD4 T cell count above 500 cells/mm(3). AIDS Res Hum Retroviruses. 2013;29(8):1161–7. 23530980 10.1089/aid.2012.0367 PMC3715811

[14] Crowell TA , Gebo KA , Blankson JN , Korthuis PT , Yehia BR , Rutstein RM , et al. Hospitalization rates and reasons among HIV elite controllers and persons with medically controlled HIV infection. J Infect Dis. 2015;211(11):1692–702. 25512624 10.1093/infdis/jiu809 PMC4447832

[15] Stafford KA , Rikhtegaran Tehrani Z , Saadat S , Ebadi M , Redfield RR , Sajadi MM . Long‐term follow‐up of elite controllers: higher risk of complications with HCV coinfection, no association with HIV disease progression. Medicine (Baltimore). 2017;96:e7348. 28658155 10.1097/MD.0000000000007348 PMC5500077

[16] Madec Y , Boufassa F , Porter K , Meyer L . Spontaneous control of viral load and CD4 cell count progression among HIV‐1 seroconverters. AIDS. 2005;19(17):2001–7. 16260907 10.1097/01.aids.0000194134.28135.cd

[17] Madec Y , Boufassa F , Porter K , Prins M , Sabin C , d'Arminio Monforte A , et al. Natural history of HIV‐control since seroconversion. AIDS. 2013;27(15):2451–60. 23912979 10.1097/01.aids.0000431945.72365.01

[18] Noel N , Lerolle N , Lecuroux C , Goujard C , Venet A , Saez‐Cirion A , et al. Immunologic and virologic progression in HIV controllers: the role of viral "Blips" and immune activation in the ANRS CO21 CODEX study. PLoS One. 2015;10:e0131922. 26146823 10.1371/journal.pone.0131922 PMC4493076

[19] Olson AD , Meyer L , Prins M , Thiebaut R , Gurdasani D , Guiguet M , et al. An evaluation of HIV elite controller definitions within a large seroconverter cohort collaboration. PLoS One. 2014;9:e86719. 24489776 10.1371/journal.pone.0086719 PMC3904947

[20] Gurdasani D , Iles L , Dillon DG , Young EH , Olson AD , Naranbhai V , et al. A systematic review of definitions of extreme phenotypes of HIV control and progression. AIDS. 2014;28(2):149–62. 24149086 10.1097/QAD.0000000000000049 PMC3882304

[21] Lambotte O , Boufassa F , Madec Y , Nguyen A , Goujard C , Meyer L , et al. HIV controllers: a homogeneous group of HIV‐1‐infected patients with spontaneous control of viral replication. Clin Infect Dis. 2005;41(7):1053–6. 16142675 10.1086/433188

[22] Ake JA , Schuetz A , Pegu P , Wieczorek L , Eller MA , Kibuuka H , et al. Safety and immunogenicity of PENNVAX‐G DNA prime administered by biojector 2000 or CELLECTRA Electroporation device with modified vaccinia ankara‐CMDR boost. J Infect Dis. 2017;216(9):1080–90. 28968759 10.1093/infdis/jix456 PMC5853809

[23] Alatrakchi N , Di Martino V , Thibault V , Benhamou Y , Katlama C , Poynard T , et al. Decreased frequencies of virus‐specific T helper type 1 cells during interferon alpha plus ribavirin treatment in HIV‐hepatitis C virus co‐infection. Aids. 2004;18(1):121–3. 15090839 10.1097/00002030-200401020-00015

